# Evaluation of a partial ring design for the INSERT SPECT/MRI system

**DOI:** 10.1186/2197-7364-2-S1-A47

**Published:** 2015-05-18

**Authors:** Kell Erlandsson, Debora Slavado, Alexandre Bousse, Brian Hutton

**Affiliations:** Institute of Nuclear Medicine, University College London, UK; The Centre for Medical Radiation Physics at the University of Wollongong, Northfields Ave, Wollongong, Australia

The aim of the INSERT project is to develop a SPECT insert for a commercial MRI system, for performing simultaneous SPECT/MRI brain studies in humans. We have previously investigated various design options for the detector system, based on a complete ring of detectors. We are now considering a partial ring, due to space limitations. We have investigated the degradation in image quality with a partial ring as compared to a full ring, and the possibility of addressing the limitations by utilising MRI data during reconstruction. Noise-free data were generated by forward-projecting a cylindrical phantom with spherical inserts for a full-ring and a partial ring system, equipped with multislit- slat (MSS) and multi-pinhole (MPH) collimators. Poisson noise was added and images were reconstructed using ML-EM and MAP-EM with a smoothing prior and an anatomical prior. Contrast-recovery (CR) was calculated for the spheres in the lower part of the phantom compared to the top ones. Background CoV was also calculated. With noise-free data, CR was 77-84% for the MSS and 82-88% for the MPH partial-ring system with 400-1600 iterations. For noisy data and MAP-EM with a smoothing prior, CR was 78-80% and 81-82%, and CoV 22-28% and 26-31%, for the MSS and MPH systems, respectively. With the anatomical prior, CR was 85-89% and 87-91%, respectively. With the partial ring-systems, the transaxial resolution in the lower part of the image is reduced. The degradation is slightly larger with MSS than MPH collimators, but the MSS collimator results in a lower noise-level. Some resolution can be recovered with more iterations, but the improvement is limited when regularisation is included. The anatomical prior offers both qualitative and quantitative improvement in image quality.

